# Empirical Bayes for DCM: A Group Inversion Scheme

**DOI:** 10.3389/fnsys.2015.00164

**Published:** 2015-11-27

**Authors:** Karl Friston, Peter Zeidman, Vladimir Litvak

**Affiliations:** The Wellcome Trust Centre for Neuroimaging, University College LondonLondon, UK

**Keywords:** empirical Bayes, random effects, fixed effects, dynamic causal modeling, Bayesian model reduction, hierarchical modeling

## Abstract

This technical note considers a simple but important methodological issue in estimating effective connectivity; namely, how do we integrate measurements from multiple subjects to infer functional brain architectures that are conserved over subjects. We offer a solution to this problem that rests on a generalization of random effects analyses to Bayesian inference about nonlinear models of electrophysiological time-series data. Specifically, we present an empirical Bayesian scheme for group or hierarchical models, in the setting of dynamic causal modeling (DCM). Recent developments in approximate Bayesian inference for hierarchical models enable the efficient estimation of group effects in DCM studies of multiple trials, sessions, or subjects. This approach estimates second (e.g., between-subject) level parameters based on posterior estimates from the first (e.g., within-subject) level. Here, we use empirical priors from the second level to iteratively optimize posterior densities over parameters at the first level. The motivation for this iterative application is to finesse the local minima problem inherent in the (first level) inversion of nonlinear and ill-posed models. Effectively, the empirical priors shrink the first level parameter estimates toward the global maximum, to provide more robust and efficient estimates of within (and between-subject) effects. This paper describes the inversion scheme using a worked example based upon simulated electrophysiological responses. In a subsequent paper, we will assess its robustness and reproducibility using an empirical example.

## Introduction

This technical note describes an application of Bayesian model reduction to the inversion of hierarchical or empirical Bayesian models (Efron and Morris, 1973; Kass and Steffey, 1989). These sorts of models are used (either implicitly or explicitly) in the analysis of multisubject studies that contain both within and between subject effects (e.g., Friston et al., 1999; Woolrich et al., 2004, 2009). In neuroimaging, models of regional responses are usually linear (convolution) models at the within-subject level. Here, we consider the problem of inverting or fitting equivalent models of effective connectivity, which are necessarily nonlinear.

The contribution of this paper is to finesse the local maxima problem that attends the inversion of nonlinear dynamic causal models (DCM). In a recent paper (Friston et al., 2015) we described the use of *Bayesian model reduction* to invert a DCM for each subject in a group study and then evaluate the posterior density over group effects, using the posterior densities from subject-specific inversions. Crucially, this means that one does not have to re-fit the subject-specific models to accumulate evidence in favor of one or more models from successive subjects. This application can be regarded as a generalization of the standard summary statistic approach; however, instead of just using point estimators as summaries of first (within-subject) level effects, one can take the full posterior density to the second (between-subject) level. Here, we apply this *empirical Bayes* procedure recursively, replacing the original priors used at the first level with empirical priors from the hierarchical inversion. In principle, with exact Bayesian inference and a convex (variational free energy) objective function, this scheme should converge after the first iteration to the (global) maximum. However, we conjectured that posterior uncertainty would decrease with successive iterations as estimators are pulled (shrunk) toward the global maximum and away from local maxima. In this paper, we verify this conjecture using simulations of realistic event related potentials and illustrate its application.

We also take the opportunity to compare and contrast Bayesian model comparison (and inference about model parameters like effective connectivity and synaptic time constants), when using empirical Bayesian estimators of group effects, relative to the more conventional modeling of the grand mean response over all subjects. In brief, we found that the two approaches were remarkably consistent; however, the empirical Bayes estimators were more efficient. We imagine that this iterative application of empirical Bayes will be useful when trying to estimate subject-specific parameters for subsequent analysis at the between-subject (or trial) level using classical or Bayesian inference at the second level. We will illustrate the latter by trying to recover the changes in connectivity responsible for condition-specific (mismatch negativity) effects.

This note comprises two sections. The first reviews the basic theory behind the iterative empirical Bayesian scheme, while the second describes the modeling of data from multiple subjects using the grand mean response and empirical Bayes. This worked example was chosen to be representative of real DCM studies—so that the procedures could be illustrated in a pragmatic way. We will therefore refer to specific (Matlab) routines that implement the procedures. These routines are part of the academic SPM software available from http://www.fil.ion.ucl.ac/spm.

## Methods and theory

Bayesian model reduction refers to the analytic inversion of reduced models using the posterior densities of a full model. Reduced models are formed from full models by removing parameters from a full model using very precise priors that shrink their values to zero. In other words, the difference between a reduced (or restricted) model and the full (or parent) model is specified in terms of their respective priors, where the reduced prior eliminates or applies shrinkage priors to combinations of model parameters. Please see (Friston and Penny, 2011) and the Appendix for a more complete discussion of Bayesian model reduction and the optimization of models. In short, Bayesian model reduction is an efficient way of estimating the posterior density one would have obtained under new priors, from the posterior of a full model.

Model reduction can be especially useful in hierarchical model inversion, where the reduced prior over the parameters of lower levels is provided by an empirical prior from the level above. For example, consider the empirical Bayes model:

(1) $$\begin{array}{l} \ln p(y,\theta^{\left(1\right)},\theta^{\left(2\right)})=\ln p(y|\theta^{\left(1\right)})+\ln p(\theta^{\left(1\right)}|\theta^{\left(2\right)})+\ln p(\theta^{\left(2\right)})\\ \;p(y|\theta^{\left(1\right)})=N(\Gamma (\theta^{\left(1\right)}),\Sigma (\theta^{\left(1\right)}))\\ \;p(\theta^{\left(1\right)}|\theta^{\left(2\right)})=N(\Gamma (\theta^{\left(2\right)}),\Sigma (\theta^{\left(2\right)}))\\ \;p(\theta^{\left(2\right)})=N(\eta ,\Sigma ) \end{array}$$

Here, Γ(θ) are (possibly nonlinear) mappings from the model parameters to the response *y* or the parameters at a lower level. Gaussian assumptions about random effects, with parameterized covariances Σ(θ), complete the specification of the likelihood model at the first level and empirical priors at subsequent levels. The ensuing generative model provides a complete probabilistic mapping from model parameters to observed data. Inference corresponds to the inversion of this mapping; from data to parameters. Usually, this inversion uses some form of approximate Bayesian inference.

Approximate Bayesian inference can always be cast as maximizing the (negative) variational free energy with respect to the sufficient statistics $\tilde{q}$ of an approximate posterior $q\left(\theta |\tilde{q}\right)$: see (Roweis and Ghahramani, 1999; Friston, 2008) for a fuller discussion. Using $\tilde{p}=\left(\eta ,\Sigma \right)$ for the sufficient statistics of a prior, this maximization can be expressed in terms of free energies at the first and second levels, where (under the Laplace approximation):

(2) $$\begin{array}{l} \;{\tilde{q}}^{(1)*}=\arg\max_{{\overset{⌢}{q}}^{\left(1\right)}}F^{\left(1\right)}({\tilde{p}}_{F},{\tilde{q}}^{\left(1\right)})\\ \;{\tilde{q}}^{(2)*}=\arg\max_{{\overset{⌢}{q}}^{\left(2\right)}}F^{\left(2\right)}({\tilde{p}}^{\left(2\right)},{\tilde{q}}^{\left(2\right)},{\tilde{q}}^{(1)*})\\ F^{\left(1\right)}({\tilde{p}}^{\left(1\right)},{\tilde{q}}^{\left(1\right)})=E_{q^{\left(1\right)}}[\ln p(y|\theta^{\left(1\right)},m)]\\ \;-\;D_{KL}[q(\theta^{\left(1\right)}|{\tilde{q}}^{\left(1\right)})||p(\theta^{\left(1\right)}|{\tilde{p}}^{\left(1\right)})]\\ F^{\left(2\right)}({\tilde{p}}^{\left(2\right)},{\tilde{q}}^{\left(2\right)},{\tilde{q}}^{\left(1\right)})=F^{\left(1\right)}({\tilde{p}}^{\left(1\right)})\\ \;-\;D_{KL}[q(\theta^{\left(2\right)}|{\tilde{q}}^{\left(2\right)})||p(\theta^{\left(2\right)}|{\tilde{p}}^{\left(2\right)})]\\ \;{\tilde{p}}^{\left(1\right)}=(\Gamma (\eta^{\left(2\right)}),\Sigma (\eta^{\left(2\right)})) \end{array}$$

In this formulation, the approximate posterior over first level parameters is evaluated in the usual way using uninformative (full) priors; for example, by inverting a DCM for each subject. Following this, the empirical prior over second (e.g., between-subject) level parameters can be evaluated by maximizing a (second level) free energy. This free energy comprises the (reduced) free energy from the first level and the complexity attributable to the empirical prior. Crucially, the reduced free energy is a function of the empirical prior and the full posterior over the first level parameters. This means we do not have to repeat the inversion at the first (e.g., within-subject) level, every time the empirical prior is updated. A more detailed explanation of this scheme can be found in the Appendix.

This scheme is the basis of the empirical Bayesian model reduction for group studies described in Friston et al. (2015). Effectively, it enables one to perform Bayesian model comparison and make inferences at the group level, without having to re-estimate each subject's DCM. In this paper, we consider the iterative optimization of (approximate) posteriors in Equation (2). In other words, we return to the first level, replacing the full prior with the empirical prior. Algorithmically, this corresponds to:

*Initialise empirical prior*

$$\begin{array}{l} {\tilde{p}}^{\left(1\right)}={\tilde{p}}_{F} \end{array}$$

*Iterate until convergence:*

(3) $$\begin{array}{l} \begin{array}{lll} {\tilde{q}}^{\left(1\right)*} & = & \arg\max_{{\overset{⌢}{q}}^{\left(1\right)}}F^{\left(1\right)}\left({\tilde{p}}^{\left(1\right)},{\overset{⌢}{q}}^{\left(1\right)}\right)\\ {\tilde{q}}^{\left(2\right)*} & = & \arg\max_{{\overset{⌢}{q}}^{\left(2\right)}}F^{\left(2\right)}\left({\tilde{p}}^{\left(2\right)},{\overset{⌢}{q}}^{\left(2\right)},{\tilde{q}}^{\left(1\right)*}\right)\\ {\tilde{p}}^{\left(1\right)} & = & \left(\Gamma \left(\eta^{\left(2\right)}\right),\Sigma \left(\eta^{\left(2\right)}\right)\right) \end{array} \end{array}$$

If we were performing exact Bayesian inference (or if our model was linear) this scheme would converge after a single iteration. This is because exact inversion under a (reduced) empirical prior produces the same results as a reduced posterior using Bayesian model reduction (see **DEMO_GROUP_PEB.m** for a numerical illustration of this). However, with nonlinear models the iteration of Equation (3) could improve the estimates, if the empirical priors are sufficiently precise to preclude local maxima in the original inversion under full priors. In other words, empirical priors can shrink the estimators toward the global maxima, providing more consistent estimators over subjects and further shrinking the empirical priors. One might therefore conjecture that iterative inversion under empirical priors might progressively eliminate local maxima—and increase model evidence (or precision at the second level). In the next section, we provide a numerical proof of principle that iterative Bayesian model reduction can indeed improve model inversion (i.e., system identification), when applied to weakly nonlinear models.

## A worked example

This section provides a worked example of how to apply empirical Bayesian reduction to multi-subject DCM studies. We consider the simplest situation, in which a group of normal subjects have been studied to characterize the functional anatomy of some cognitive process. Our objective is therefore to combine data from several subjects to optimize Bayesian model comparison (to test specific hypotheses changes in effective connectivity) and Bayesian model averaging (to identify and quantify condition-specific connectivity changes that have been induced experimentally).

**Figure 1:**
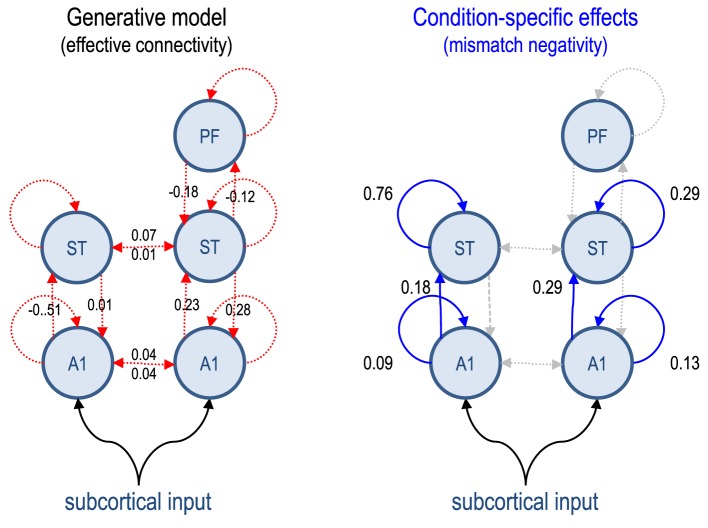
**This figure describes the generative model used to simulated data for subsequent model inversion and reduction**. The circles represent electromagnetic sources of data (recorded by 128 sensors or channels). These sources have intrinsic dynamics, modeled with eight ordinary differential equations per source. The dynamics are perturbed with a parameterized exogenous (subcortical) input and coupled to each other through (effective) connections. Because the sources are organized hierarchically, one can refer to (between-source or extrinsic) connections as forward or backward. The strengths of these connections correspond to the key model parameters with random effects. The group averages (based on analysis of grand mean data from normal subjects) are shown alongside their connection: red connections (left panel) indicate connections that are common to both conditions, while blue connections (right panel) are equipped with parameters encoding condition-specific effects. The values are log scale parameters, therefore a negative value means a smaller (excitatory) connection strength. A1, primary auditory source; ST, superior temporal source; PF, prefrontal source.

We chose a fairly standard setup to reproduce the sorts of data and questions typically encountered in DCM studies. We simulated data from 16 subjects based on a previously reported EEG study of the mismatch negativity (Garrido et al., 2007)—a paradigm that has been examined extensively using DCM in both normal subjects and schizophrenia (Garrido et al., 2009a; Fogelson et al., 2014). In brief, subjects are presented with streams of auditory tones, whose frequency is changed sporadically and unexpectedly. These correspond to *standard* and *oddball* stimuli, which evoke responses that can be recorded electromagnetically (here with EEG) as event related potentials. Previous studies have established a minimal network of five cortical sources is sufficient to explain the evoked responses, where differences between standard and oddball stimuli can be accounted for by differences in connectivity both within (intrinsic) and between (extrinsic) sources (Garrido et al., 2009b), see Figure 1.

We generated data for each of the 16 subjects using the locations of five sources (right and left auditory sources, right and left superior temporal sources, and a right prefrontal source) and the (connectivity) parameters estimated from a previously reported DCM study of the grand mean response (Garrido et al., 2007). The parameters used to generate the simulated data were obtained by inverting a model with hierarchical connectivity among the five sources, while allowing for condition-specific changes in the intrinsic connectivity of the auditory and temporal sources and extrinsic connectivity from the auditory to temporal sources. Physiologically, this corresponds to a change in the excitability of neuronal populations in the auditory and temporal sources, with a selective change in forward connectivity (i.e., temporal sensitivity to ascending auditory input). We deliberately precluded changes in backward connections to see whether model inversion could correctly identify changes in, and only in, forward connections.

The resulting estimates were used as group mean values, to which random Gaussian variates were added to produce subject-specific parameters. These random effects were sampled from the prior distribution over model parameters, described in Garrido et al. (2009a). More precisely, we fixed the between-subject parametric variability to be a sixteenth of the usual prior variances used in this sort of DCM. These prior variances now play the role of full priors on the second level (group mean) parameters. This model has 158 neuronal parameters and 40 spatial parameters for each subject. Of these, we were particularly interested in 18 parameters encoding the strength of connections within and between sources and how they changed with experimental condition (see Figure 1). We generated event related potentials using the lead field employed in the empirical study but with random (dipole orientation and amplitude) parameters sampled from their prior distribution.

**Figure 2:**
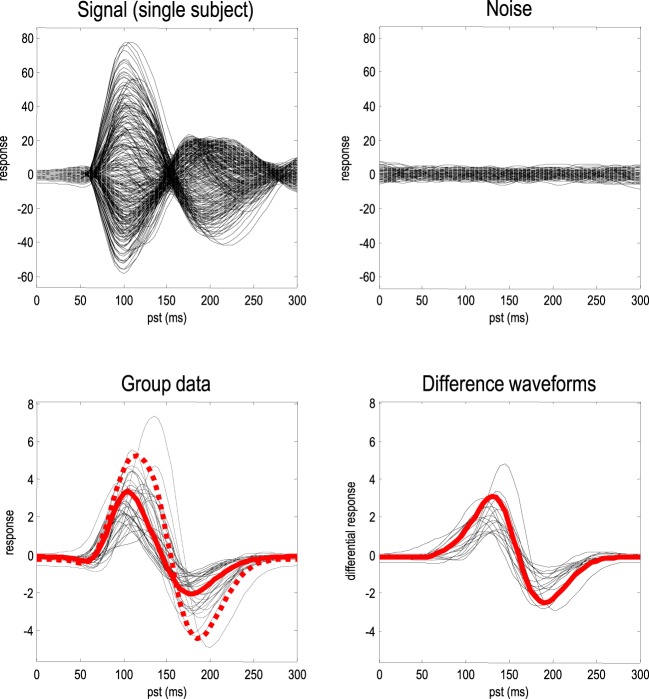
**Simulated data**. The upper panel shows channel data from a single subject as a function of peristimulus time. The solid lines (upper left) correspond to the simulated signal, while the dotted lines correspond to signal plus noise. For comparison, the observation noise is shown on the upper right. The lower panels show simulated responses over subjects in terms of a mixture of sensor data based upon the first principal component of the prior predictive covariance. The solid lines (middle left) correspond to the first (standard) condition, while the dotted lines report the second (oddball) condition. The right panel shows the condition-specific effects in terms of the waveform differences between the two conditions; namely, the mismatch negativity. The red lines report the grand means of the condition-specific responses (left) and differences (right).

Figure 2, shows an example of the data simulated over 128 channels. The upper left panel shows the simulated sensor data with and without simulated observation noise. Observation or sensor noise was created by convolving Gaussian noise with a smoothing kernel of eight (4 ms) time bins. The observation noise was scaled to have a standard deviation that was an eighth of the simulated signal. This signal-to-noise ratio corresponds roughly to the levels that one would expect after averaging 64 trials, where the amplitudes of signal and noise were equivalent. The lower panels show the between-subject variance in terms of the response of the first principal component over channel space; for the two conditions (left panel) and the condition-specific differences or mismatch negativity (right panel).

### Empirical bayesian inversion and model comparison

**Figure 3:**
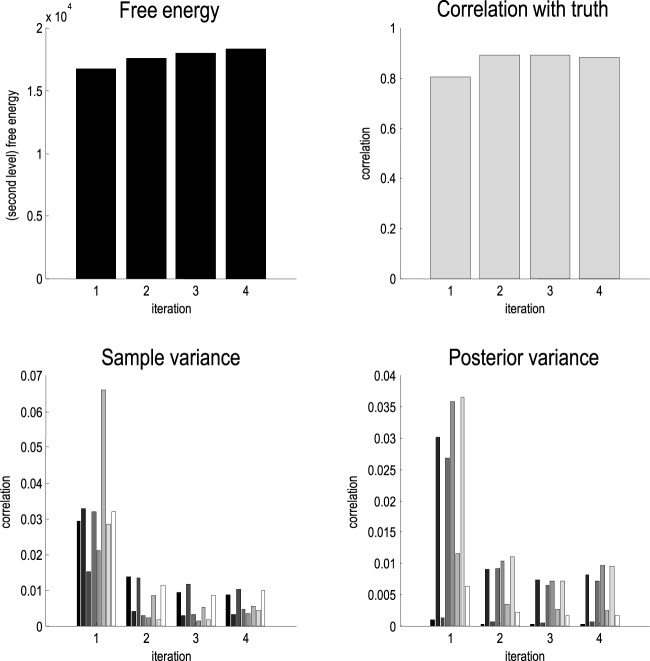
**Upper panels:** The (second level or total) free energy (left) and correlations with true parameter values (right) over iterations of empirical Bayesian inversion of the simulated data. In the absence of local maxima and other convergence failures, this free energy would otherwise saturate after the first iteration. **Lower panels**: these report the sample and posterior variance of the interesting (condition specific) scaling effects, averaged over subjects. These variances fall markedly after the second iteration and then remain relatively stable.

We inverted the data from each subject using iterative empirical Bayesian inversion (as described in Equation (3) and implemented in **spm_dcm_peb_fit.m**). In this case, the second level model was a simple general linear model with a mean or constant term for each parameter at the first level. Figure 3 shows the free energy continues to increase after the first iteration. As noted above, this and subsequent increases can only be explained by local maxima (or other convergence behavior) induced by the non-linear nature of our DCM. If our model was linear and the free energy objective function was perfectly behaved, then the free energy would not change with successive iterations. The correlations between the subject-specific parameter estimates and the true values are also shown in Figure 3. In line with the local maxima conjecture, the correlations increase after the first iteration and then remain relatively high (at about 0.88). The improvement in the correlations is mirrored by a decrease in the sample and posterior variance of the parameters—as shown for the changes in connectivity in the lower panels of Figure 3.

Note that the correlation with the true values falls very slightly after the fourth iteration. This behavior is characteristic of the (extensive) simulations that we have performed. Generally, the free energy continues to increase with successive iterations but after a few iterations, the correlations with the true values tend to decrease, and the posterior covariance starts to increase. We therefore require the (log determinant) of the posterior covariance to decrease before terminating the iterations. The rationale for this convergence criterion is that if the empirical priors are destroying local maxima, we would expect the posterior variance to decrease as subject-specific estimators converge to their global minima, which we suppose are in the vicinity of the group mean. Practically, this means the iterative scheme usually converges after three or four iterations.

**Figure 4:**
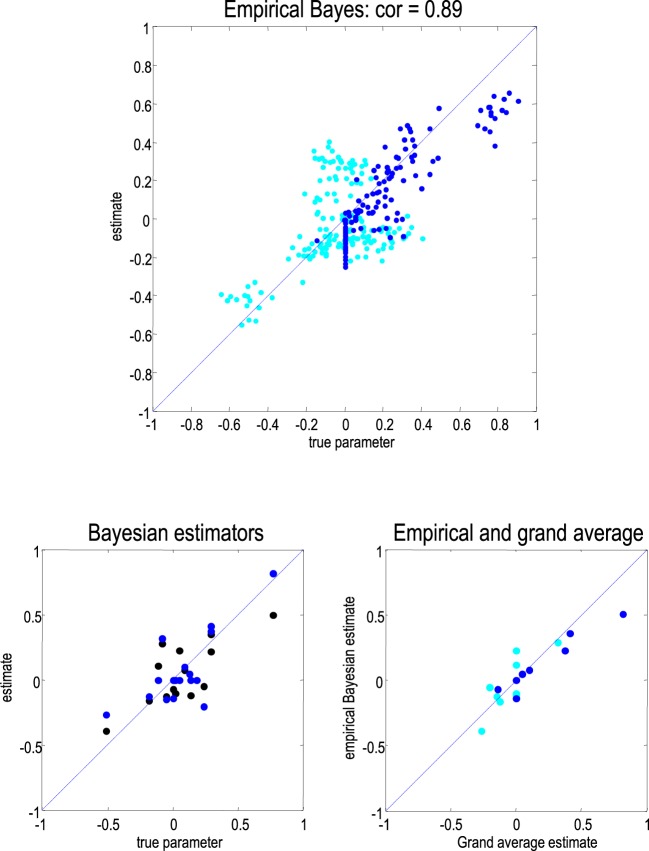
**Upper panel:** A plot of the empirical Bayesian estimators of connections (cyan) and their condition-specific changes (blue) against the true values, over all subjects. The correlation coefficient corresponds to the condition-specific changes and is remarkably high. **Lower left panel**: this shows the posterior estimates of parameter averages from the empirical Bayesian analysis (black) and analysis of the grand mean (blue), plotted against the true group mean values. **Lower right panel**: this plots the two estimators against each other. As above, cyan denotes connections strengths *per se*, while blue corresponds to changes underlying the mismatch negativity.

The correlation between the empirical Bayesian estimators and the true parameter values (over subjects) are shown in Figure 4 (upper panel). At these, not unrealistic, levels of noise and intersubject variability, there is a remarkably high correlation (of 0.89) between the parameters of interest; namely, those responsible for condition-specific effects (blue dots). A key thing to take from the results in Figure 4 is that the dispersion of estimators for any given parameter is very similar to the true dispersion. This suggests that the between-subject variability has been estimated reasonably accurately, thereby enabling optimal shrinkage to the group means. Note the vertical line of blue dots that correspond to the (backward) extrinsic connections that we fixed to their prior mean (with a log scaling of zero). Although the true values were zero, the empirical Bayesian estimators suggest these connections were actually reduced very slightly during the oddball conditions. We now look at the parameter estimates in greater detail, at the between-subject level:

Having inverted the group data, we then applied Bayesian model reduction to evaluate the Bayesian model average over the group means. In this example, we performed an exhaustive search over all combinations of parameters and their condition-specific changes; using **spm_dcm_peb.m** to evaluate the posterior densities over second level parameters and **spm_dcm_peb_bmc.m** for Bayesian model averaging. The ensuing posterior densities are shown before and after Bayesian model reduction in the right panels of Figure 4. Crucially, every condition-specific increase was detected with a high degree of posterior confidence, with one exception (one of the forward connections). Conversely, the connections that did not change were estimated to decrease slightly—although the posterior confidence interval on one of the backward connections included zero. The lower right panel of Figure 4 shows the results of Bayesian model comparison, when comparing models that did and did not include each parameter. This is a simple form of Bayesian model comparison that quantifies the belief that each parameter deviated from its prior expectations.

### Analysis of the grand average

**Figure 5:**
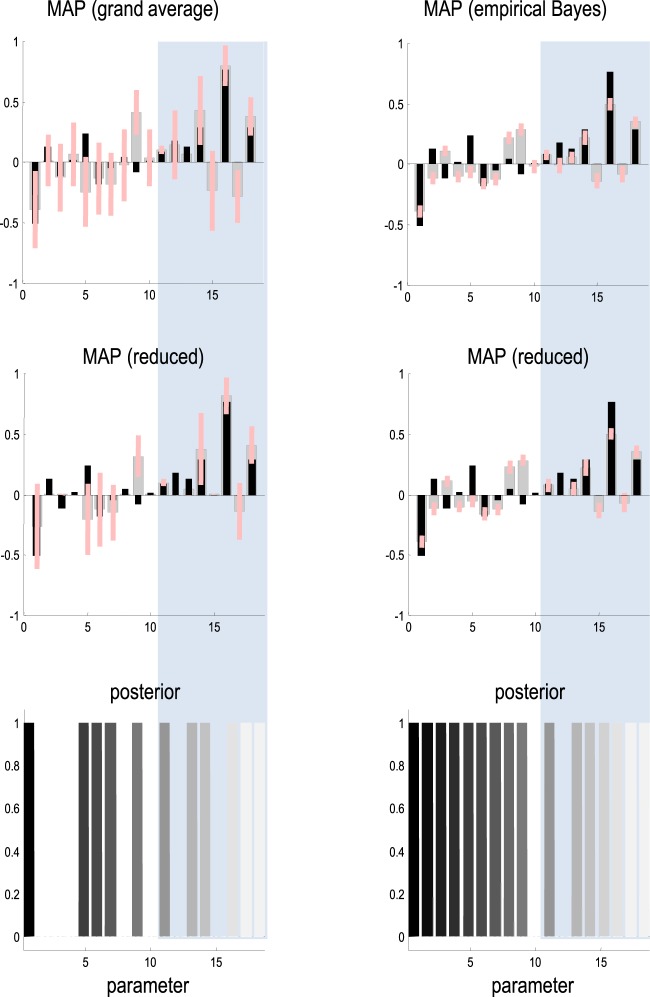
**This figure compares the posterior estimates of group means based upon the grand average (left panels) and the empirical Bayesian estimators (right panels)**. The top row shows the estimates prior to model reduction, while the second row shows the same results after redundant parameters have been eliminated. The gray bars correspond to conditional expectations, while the pink bars denote 90% Bayesian confidence intervals. These are superimposed on the true values, shown as black bars. The first 10 parameters correspond to the underlying connectivity (right panel in Figure 2), while the final eight parameters (in the shaded regions) correspond to condition specific changes mediating the mismatch negativity (left panel in Figure 2). The lower row reports the posterior probability of models containing each parameter, compared to models that do not (following an exhaustive model search over all combinations of the reported parameters).

Finally, we repeated the above Bayesian model averaging following inversion of the grand mean data. These simulated grand mean data were obtained by averaging the responses over the 16 subjects, which were subsequently inverted in the usual way (using **spm_dcm_fit.m**). Bayesian model reduction and averaging (using **spm_dcm_bmr.m**) produced results that were remarkably similar to the second level group means obtained with empirical Bayesian inversion. The lower panel of Figure 5 illustrates this correspondence by plotting the Bayesian model averages following empirical Bayesian inversion against the Bayesian model averages obtained by inverting the average of the data.

This is a reassuring result because it means similar inferences can be obtained by inverting the average data over subjects and a full (empirical Bayesian) analysis. This is important because previous group studies using DCM have used the grand mean (average) responses. This sort of correspondence is nontrivial because the model is nonlinear. In other words, the parameters estimated from average of the data are not necessarily the average of the parameters estimated from the data. Having said this, the correspondence shown in Figure 5 probably owes much to the linearity of the mapping between neuronal sources and measured data, implicit in a linear electromagnetic forward model—that is part of the DCM for event related potentials (ERP).

The posterior densities before and after Bayesian model averaging are shown in Figure 5 (left panels) for comparison with the corresponding empirical Bayesian estimates. The key thing to note here is that the posterior confidence intervals are larger for the grand average analysis. In other words, the empirical Bayesian scheme provides more precise estimates. This has two consequences. First, the empirical Bayesian estimators are biased toward the prior mean and generally underestimate the true effects, in relation to the equivalent estimators based upon the grand average. However, the greater uncertainty associated with the grand average estimators means that several connections are eliminated following Bayesian model reduction. This effect is particularly severe for the intrinsic and extrinsic connections at the lowest level of the auditory hierarchy (compare the results of Bayesian model comparison in the lower panels in Figure 4).

## Conclusion

In conclusion, we have described an iterative extension to an empirical Bayesian scheme for nonlinear models. By repeatedly inverting models at the first level, under empirical priors furnished by higher levels, one can finesse the local maxima problem by shrinking estimators to the global mean. This provides more robust estimates at the first (e.g., within-subject) level for subsequent Bayesian model comparison and inferences about key model parameters. Furthermore, we have shown that, in principle, similar results can be obtained when analysing the grand mean data from ERP studies; however, ERP studies may be a rather special case given the linear relationship between (hidden) neuronal responses and observed data (and identical paradigms over subjects).

This iterative application of empirical Bayes should provide more efficient and accurate estimates of parameters obtained from multiple trials, sessions or subjects. Although we have focused on Bayesian inference about group means in this technical note, subject-specific estimators can be treated as summary statistics in the usual way—and used to test for particular between trial or subject effects using classical inference. However, there is a caveat here: because these estimates enjoy the benefit of empirical shrinkage priors, they will all be shrunk to the group mean. This means that classical tests against the null hypothesis of a mean of zero will be biased. This does not affect any other tests; for example, the effects of age or other parametric (between trial or subject) variables.

We are currently evaluating the reproducibility and robustness of this iterative scheme using empirical data. Key questions here include reproducibility in terms of inference about models and parameters under different models, different data and the difference between empirical Bayesian averaging vs. inversion of the grand average.

Future work will focus on the convergence of our iterative scheme for group data. This is an interesting problem (highlighted by our reviewers), because the very presence of local minima—and related violations of the Laplace assumption—means that variational free energy could be a poor approximation to model evidence. In other words, the problem we are trying to finesse precludes a simple free energy optimization (indeed, one occasionally sees decreases in free energy with successive iterations). This is further compounded by the fact that approximate Bayesian inference based upon variational free energy is known to provide overconfident solutions, even when the assumptions of the Laplace approximation are met. Our (pragmatic) solution to this is to use the reduction in posterior uncertainty at the between subject level as a criterion for convergence. The rationale here is that shrinkage to the group mean will be reflected in a more precise posterior density (as local minima are destroyed). This appears to work for a variety of imaging modalities and data features; however, a full validation of this criterion would require (multimodal) posterior distributions that eschew the Laplace assumption. These can be accessed using sampling schemes that are the focus of current research (e.g., Sengupta et al., 2014). An alternative approach could call on extensions of Variational Bayes and mean-field approximation schemes, via the use of mixture distributions (e.g., Jaakkola and Jordan, 1998). Essentially, instead of selecting one particular mean-field solution, these schemes form a weighted average (a mixture) of several mean-field solutions. Crucially, they rely on the assumption that minima are not close to each other, which might not be a completely unrealistic assumption in the context of DCM.

## Software note

The procedures described in this paper are implemented as Matlab routines in the SPM software (http://www.fil.ion.ucl.ac/spm). The key routines are **spm_dcm_peb_fit.m**, **spm_dcm_peb.m** and **spm_dcm_peb_bmc.m**. These (annotated) routines include a help section for further guidance on their functionality. A demonstration of their use is provided in the DEM Toolbox (invoked by typing DEM). The code can be edited and executed by selecting the “PEB with BMR” button or running the following routines directly: **DEMO_BMR_PEB.m** and **DEMO_GROUP_PEB.m**.

### Conflict of interest statement

The authors declare that the research was conducted in the absence of any commercial or financial relationships that could be construed as a potential conflict of interest.

## Appendix

### Bayesian model inversion

Bayesian model reduction refers to the Bayesian inversion of reduced models using the posterior density of a full model. Consider a generative model specified in terms of its likelihood and priors. For example, a model with additive Gaussian noise:

(A1) $$\begin{array}{l} \ln p\left(y,\theta^{\left(1\right)},\theta^{\left(2\right)}\right)=\ln p(y|\theta^{\left(1\right)})+\ln p(\theta^{\left(1\right)}|\theta^{\left(2\right)})+\ln p(\theta^{\left(2\right)})\\ p\left(y|\theta^{\left(1\right)}\right)=N(\Gamma (\theta^{\left(1\right)}),\Sigma (\theta^{\left(1\right)}))\\ p\left(\theta^{\left(1\right)}|\theta^{\left(2\right)}\right)=N(\Gamma (\theta^{\left(2\right)}),\Sigma (\theta^{\left(2\right)}))\\ p\left(\theta^{\left(2\right)}\right)=N(\eta ,\Sigma ) \end{array}$$

Here, Γ(θ) is a mapping from the parameters of a model to the predicted response *y*. Gaussian assumptions about observation noise, with a parameterized covariance Σ(θ), define the likelihood model that, when equipped with (Gaussian) priors, specifies the generative model. Having specified in the generative model, one can now estimate the sufficient statistics of the approximate posterior and associated free energy by solving:

(A2) $$\begin{array}{l} \;{\tilde{q}}^{*}=\arg\max_{\overset{⌢}{q}}F(\tilde{p},\tilde{q})\\ F(\tilde{p},\tilde{q})=\underset{\text{accuracy}}{\underset{︸}{E_{q}[\ln p(y|\theta )]}}-\underset{\text{complexity}}{\underset{︸}{D_{KL}[q(\theta |\tilde{q})||p(\theta |\tilde{p})]}}\\ \;q\left(\theta |{\tilde{q}}^{*}\right)\approx p(\theta |y,\tilde{p})\\ \;F(\tilde{p},{\tilde{q}}^{*})\approx \ln p(y|\tilde{p}) \end{array}$$

### Bayesian model reduction

Now we want to estimate the posterior under a new model after eliminating some parameters to produce a reduced model. Consider Bayes rule for reduced and full models (*m*_*R*_, *m*_*F*_):

(A3) $$\begin{array}{lll} p\left(\theta |y,m_{R}\right)p\left(y|m_{R}\right) & =p\left(y|\theta ,m_{R}\right)p\left(\theta |m_{R}\right)\; & \;\\ p\left(\theta |y,m_{F}\right)p\left(y|m_{F}\right) & =p\left(y|\theta ,m_{F}\right)p\left(\theta |m_{F}\right)\; & \; \end{array}$$

If the models differ only in terms of their priors, then the likelihoods are identical and Equation (A3) can be simplified:

(A4) $$\begin{array}{rcl} p\left(y|\theta ,m_{R}\right) & = & p\left(y|\theta ,m_{F}\right)\Rightarrow \frac{p\left(\theta |m_{R}\right)}{p\left(\theta |m_{F}\right)}=\frac{p\left(\theta |y,m_{R}\right)p\left(y|m_{R}\right)}{p\left(\theta |y,m_{F}\right)p\left(y|m_{F}\right)}\Rightarrow \\ p\left(\theta |y,m_{R}\right) & = & p\left(\theta |y,m_{F}\right)\frac{p\left(\theta |m_{R}\right)}{p\left(\theta |m_{F}\right)}\frac{p\left(y|m_{F}\right)}{p\left(y|m_{R}\right)}\\ \frac{p\left(y|m_{R}\right)}{p\left(y|m_{F}\right)} & = & \int p\left(\theta |y,m_{F}\right)\frac{p\left(\theta |m_{R}\right)}{p\left(\theta |m_{F}\right)}d\theta \end{array}$$

This provides the posterior distribution over the parameters of the reduced model and (by integrating over the parameters) the evidence ratio of the reduced and full models. Substituting the approximate posterior $q\left(\theta |{\tilde{q}}^{*}\right)$ and model evidence $F\left(\tilde{p},{\tilde{q}}^{*}\right)$ from Equation (A2) and replacing models (*m*_*R*_, *m*_*F*_)with the sufficient statistics of their priors $\left({\tilde{p}}_{R},{\tilde{p}}_{F}\right)$ we get:

(A5) $$\begin{array}{r} q\left(\theta |{\tilde{q}}_{R}^{*}\right)\approx q\left(\theta |{\tilde{q}}_{F}^{*}\right)\frac{p\left(\theta |{\tilde{p}}_{R}\right)}{p\left(\theta |{\tilde{p}}_{F}\right)}\frac{p\left(y|{\tilde{p}}_{F}\right)}{p\left(y|{\tilde{p}}_{R}\right)}\\ F\left({\tilde{p}}_{R},{\tilde{q}}_{R}^{*}\right)\approx \ln\;\int q\left(\theta |{\tilde{q}}_{F}^{*}\right)\frac{p\left(\theta |{\tilde{p}}_{R}\right)}{p\left(\theta |{\tilde{p}}_{F}\right)}d\theta +F\left({\tilde{p}}_{F},{\tilde{q}}_{F}^{*}\right) \end{array}$$

These (approximate) equalities mean one can evaluate the posterior and evidence of any reduced model, given the posteriors of the full model.

### Parametric empirical bayes

If we now apply Equation (A4) to the hierarchical generative model in Equation (1), we can express its inversion in terms of reduced free energies [as summarized in Equation (2)]. These free energies have exactly the same forms as in Equation (A2):

(A6) $$\begin{array}{l} \;{\tilde{q}}^{(1)*}=\arg\max_{{\overset{⌢}{q}}^{1}}F^{\left(1\right)}({\tilde{p}}_{F},{\tilde{q}}^{\left(1\right)})\\ \;{\tilde{q}}^{(2)*}=\arg\max_{{\overset{⌢}{q}}^{2}}F^{\left(2\right)}({\tilde{p}}^{\left(2\right)},{\tilde{q}}^{\left(2\right)},{\tilde{q}}^{(1)*})\\ \;F^{\left(1\right)}({\tilde{p}}^{\left(1\right)},{\tilde{q}}^{\left(1\right)})=\underset{\text{accuracy}}{\underset{︸}{E_{q^{\left(1\right)}}[\ln p(y|\theta^{\left(1\right)},m)]}}-\underset{\text{1st level complexity}}{\underset{︸}{D_{KL}[q(\theta^{\left(1\right)}|{\tilde{q}}^{\left(1\right)})||p(\theta^{\left(1\right)}|{\tilde{p}}^{\left(1\right)})]}}\\ F^{\left(2\right)}({\tilde{p}}^{\left(2\right)},{\tilde{q}}^{\left(2\right)},{\tilde{q}}^{\left(1\right)})=\underset{\text{accuracy}}{\underset{︸}{E_{q^{\left(1\right)}}[\ln p(y|\theta^{\left(1\right)},m)]}}-\underset{\text{1st level complexity}}{\underset{︸}{E_{q^{\left(2\right)}}[D_{KL}[q(\theta^{\left(1\right)}|{\tilde{q}}^{\left(1\right)})||p(\theta^{\left(1\right)}|{\tilde{p}}^{\left(1\right)})]}}-\underset{\text{2nd level complexity}}{\underset{︸}{D_{KL}[q(\theta^{\left(2\right)}|{\tilde{q}}^{\left(2\right)})||p(\theta^{\left(2\right)}|{\tilde{p}}^{\left(2\right)})]}}\\ \;=\underset{\text{accuracy and 1st level complexity}}{\underset{︸}{E_{q^{\left(2\right)}}[F^{\left(1\right)}({\tilde{p}}^{\left(1\right)},{\tilde{q}}^{\left(1\right)})]}}-\underset{\text{2nd level complexity}}{\underset{︸}{D_{KL}[q(\theta^{\left(2\right)}|{\tilde{q}}^{\left(2\right)})||p(\theta^{\left(2\right)}|{\tilde{p}}^{\left(2\right)})]}}\\ \;{\tilde{p}}^{\left(1\right)}=\underset{\text{empirical prior}}{\underset{︸}{\left(\Gamma (\theta^{\left(2\right)}),\Sigma (\theta^{\left(2\right)})\right)}} \end{array}$$

This optimization problem has been written in a way that clarifies the role of reduced free energy $F^{\left(1\right)}\left({\tilde{p}}^{\left(1\right)},{\tilde{q}}^{\left(1\right)}\right)$: Here, the (full) approximate posterior is evaluated in the usual way, using relatively uninformative (full) priors. Then the approximate posterior over second level parameters is computed from a (second level) free energy that comprises the expected (reduced) free energy from the first level and the complexity attributable to the posterior over second level parameters. Crucially, this means one never needs to re-estimate the first level posterior. The expression in Equation (2) is slightly simpler than Equation (A6), because it assumes the posteriors are Gaussian (see Equation 12 in Friston et al., 2015).
